# Inflammatory Gene Expression in Livers Undergoing Ex Situ Normothermic Perfusion Is Attenuated by Leukocyte Removal From the Perfusate

**DOI:** 10.1097/TP.0000000000005214

**Published:** 2025-01-20

**Authors:** Kasra Bahadori, Colin Y.C. Lee, John R. Ferdinand, Mia Cabantous, Andrew J. Butler, Foad J. Rouhani, Christopher J.E. Watson, Menna R. Clatworthy

**Affiliations:** 1 Molecular Immunity Unit, Department of Medicine, Laboratory of Molecular Biology, University of Cambridge, Cambridge, United Kingdom.; 2 National Institute of Health Research Blood and Transplant Research Unit in Organ Donation and Transplantation, Cambridge, United Kingdom.; 3 Department of Surgery, University of Cambridge, Cambridge, United Kingdom.; 4 The Francis Crick Institute, London, United Kingdom.; 5 Cellular Genetics, Wellcome Sanger Institute, Hinxton, United Kingdom.

## Abstract

**Background.:**

Ex situ normothermic perfusion (ESNP) is a method to evaluate and potentially recondition organs before transplantation. However, increased expression of inflammatory molecules, including by tissue-resident immune cells, may occur during the perfusion process, potentially negating the beneficial effects of perfusion.

**Methods.:**

We used RNA sequencing to assess gene expression in 31 livers undergoing ESNP, including 23 donated after circulatory death (DCD) and 8 donated after brain death. In 7 DCD livers, a leucocyte filter was added to the circuit during perfusion. Biopsies were available for transcriptomic assessment in all cases at the start of perfusion and at varying time points postperfusion.

**Results.:**

During ESNP in DCD livers, we observed an increase in proinflammatory, profibrinolytic, and prorepair pathway genes. *SERPINE1*, encoding plasminogen activator inhibitor-1, was among the genes most significantly upregulated during perfusion in DCD livers, potentially promoting fibrin clot persistence in vasculature. We also found increased expression of monocyte and neutrophil recruiting chemokine and proinflammatory cytokine transcripts during ESNP, but several prorepair molecules, including thymic stromal lymphopoietin, were also upregulated. In both DCD and donation after brain death livers, interferon-gamma response genes were enriched, whereas oxidative phosphorylation genes decreased in organs with high perfusate alanine transaminase, a biomarker associated with adverse clinical outcomes. The inclusion of a leukocyte filter in the perfusion circuit mitigated the induction of inflammation/immune pathway genes during perfusion and was associated with enrichment in oxidative phosphorylation genes.

**Conclusions.:**

Leukocyte removal during ESNP abrogates transcriptional changes that are associated with unfavorable clinical outcomes, potentially benefiting human livers undergoing ESNP.

## INTRODUCTION

Liver transplantation is a life-saving treatment for patients with end-stage liver disease, but organ shortage is a major challenge. In the United Kingdom, 11% of patients listed for a liver in 2020–2021 died before receiving a transplant^1^; similarly, in the United States, despite record numbers of transplants in 2023, >1000 patients died on the waiting list.^2^ Many potentially usable livers are not transplanted, particularly livers from donors after circulatory death (DCD). Of the 1429 deceased donors in the United Kingdom in 2022–2023, only 850 livers (59%) were transplanted (78% from brain-dead donors, 38% from DCD donors); similarly, in the United States in 2022, of the 14 905 deceased donors, only 8924 (60%) livers were transplanted, with livers from DCD donors most likely to be declined or not recovered.^2,3^ Targeting these unused donor organs is necessary if waiting list mortality is to be reduced.

In recent years, initiatives to increase organ utilization have concentrated on improving the preservation of organs and providing a means of evaluating function before transplantation. These have largely focused on continuous perfusion of organs, either in the cold with oxygenated preservation solutions or at normothermia using a red cell-based perfusate. Recent randomized studies have shown the efficacy of both approaches,^4,5^ particularly in improving early allograft function. Studies using ex situ normothermic perfusion (ESNP) devices have enabled acceptable clinical outcomes from livers initially declined for transplantation by conventional criteria.^6,7^

ESNP provides a platform to assess and potentially recondition organs, thus increasing utilization.^4^ The molecular processes occurring in liver grafts during this process remain to be fully elucidated, although early studies have suggested a reduction in markers of ischemia/reperfusion injury (IRI) in recipients of such grafts.^8^ More generally, ESNP enables controlled organ reperfusion after a period of ischemia during cold storage, providing an opportunity to remove proinflammatory, cell damage–associated molecules, and donor leucocytes that can further exacerbate organ inflammation.^9^ These donor leucocytes may be trapped within the graft microcirculation or be tissue-resident passenger immune cells that mobilize into the perfusion circuit during ESNP.^10-13^ To date, there is no information on whether or how leucocyte depletion during ESNP affects the molecular pathways activated in livers during this process. Here, we sampled human livers retrieved for transplantation at the start, during, and at the end of perfusion, in the presence and absence of a leucocyte filter. We applied bulk RNA sequencing to profile cellular responses to ESNP and determine the effect of leucocyte depletion.

## MATERIALS AND METHODS

### Study Conduct

The study was conducted in accordance with both the Declarations of Helsinki and Istanbul. The study was approved by the Cambridge East research ethics committee (14/EE/0137).

### Study Subjects and Protocol

Donor livers offered for research or transplantation were recruited to the study and underwent ESNP using either Liver Assist (XVIVO, Groningen, the Netherlands) or, latterly, Metra (Organox, Oxford, United Kingdom). This included 23 DCD livers and 8 donation after brain death (DBD) livers (**Figure S1A, SDC,** http://links.lww.com/TP/D167). In 7 DCD livers, a cell filter was added to the perfusion circuit (**Figure S1A, SDC,** http://links.lww.com/TP/D167). The perfusate comprised third-party packed red cells suspended in Gelofusine (B. Braun Medical, Sheffield, United Kingdom), with additives and infusions of heparin, insulin, and epoprostenol (and bile salts with the Metra) continued throughout perfusion, as previously described.^6^ Needle-core biopsies were taken before commencing ESNP in all livers but were variably performed at the end of perfusion (**Figure S1A, SDC,** http://links.lww.com/TP/D167). Biopsies were stored in RNALater (Ambion). All samples underwent RNA extraction and sequencing. The perfusate was sampled at intervals during perfusion and centrifuged, and the supernatant (plasma) was frozen at –20 °C. Cytokine levels were measured by The Core Biochemical Assay Laboratory, Cambridge, using MesoScale Discovery Human 10-plex Chemokine and Proinflammatory Cytokine panels.

### Study Design

**FIGURE 1.**
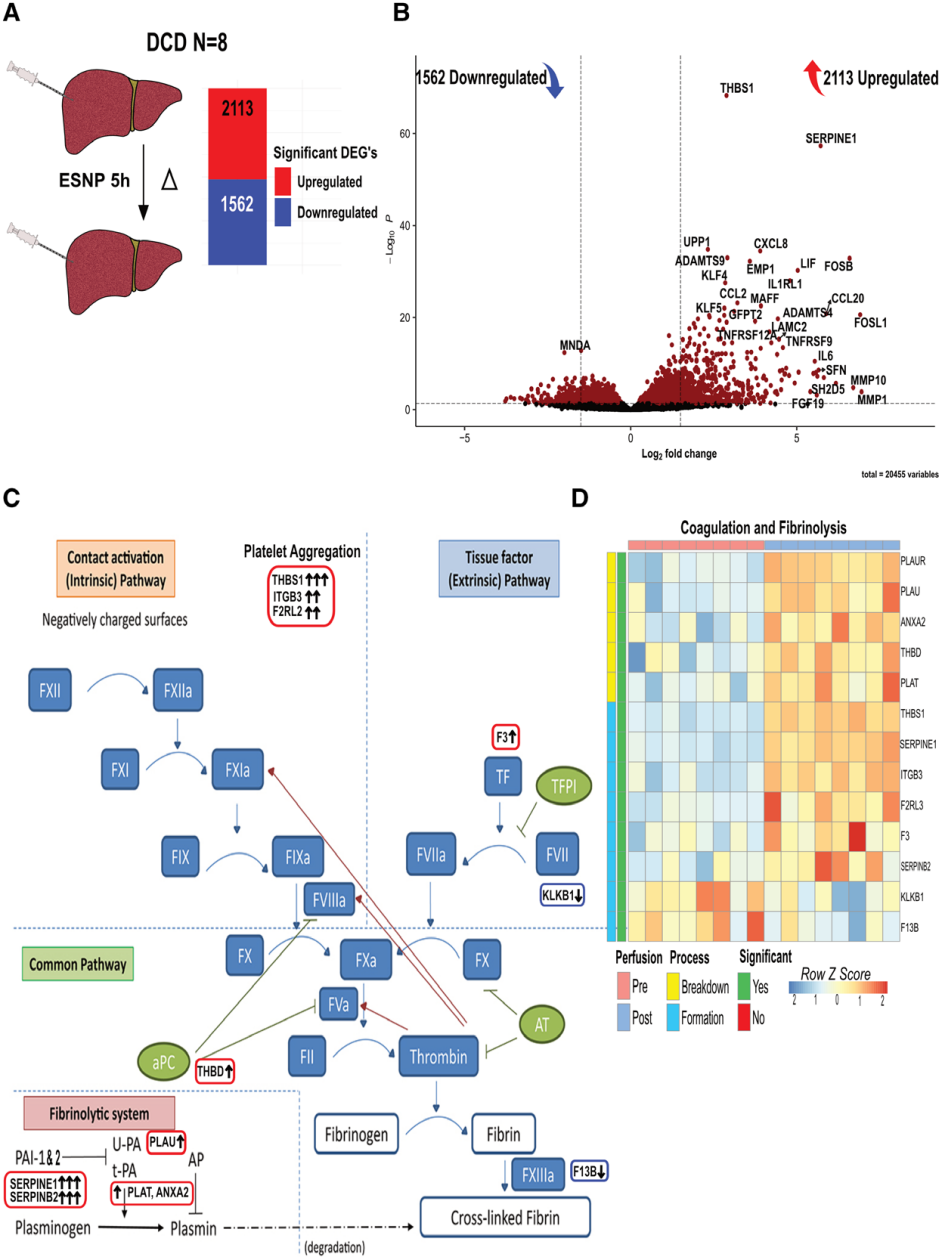
Significant changes in gene expression in livers undergoing ESNP. A, Bar chart displaying DEG of paired biopsy samples collected from 8 DCD livers both at the start and end of ESNP. Genes denoted in red indicate upregulation and those in blue represent downregulation. B, Volcano plot representing the change in gene expression after 5 h of ESNP, with the top significantly (adjusted *P* < 0.05) upregulated and downregulated genes labeled. C, Modified schematic of the coagulation and fibrinolysis pathways based on Stallone et al. (2020) in *Frontiers in Immunology*.^14^ Gene names are highlighted to show relevant trends in the experiment. The term “platelet aggregation” represents the primary hemostasis process. Genes related to clot breakdown are boxed in red, whereas those related to clot formation are boxed in blue. Arrows alongside gene names indicate the L2FC in gene expression: 1 arrow represents an L2FC >1; 2 arrows, an L2FC >2; and 3 arrows, an L2FC >3. D, Heatmap of clustered genes related to the coagulation and fibrinolysis pathways expressed post-ESNP, all genes exhibit a significant differential expression (adjusted *P* < 0.05) and are highlighted in green with their relevant role in the coagulation process highlighted in yellow (breakdown) or teal (formation). DCD, donation after circulatory death; DEG, differentially expressed gene; ESNP, ex situ normothermic perfusion; L2FC, log2 fold change.

**FIGURE 2.**
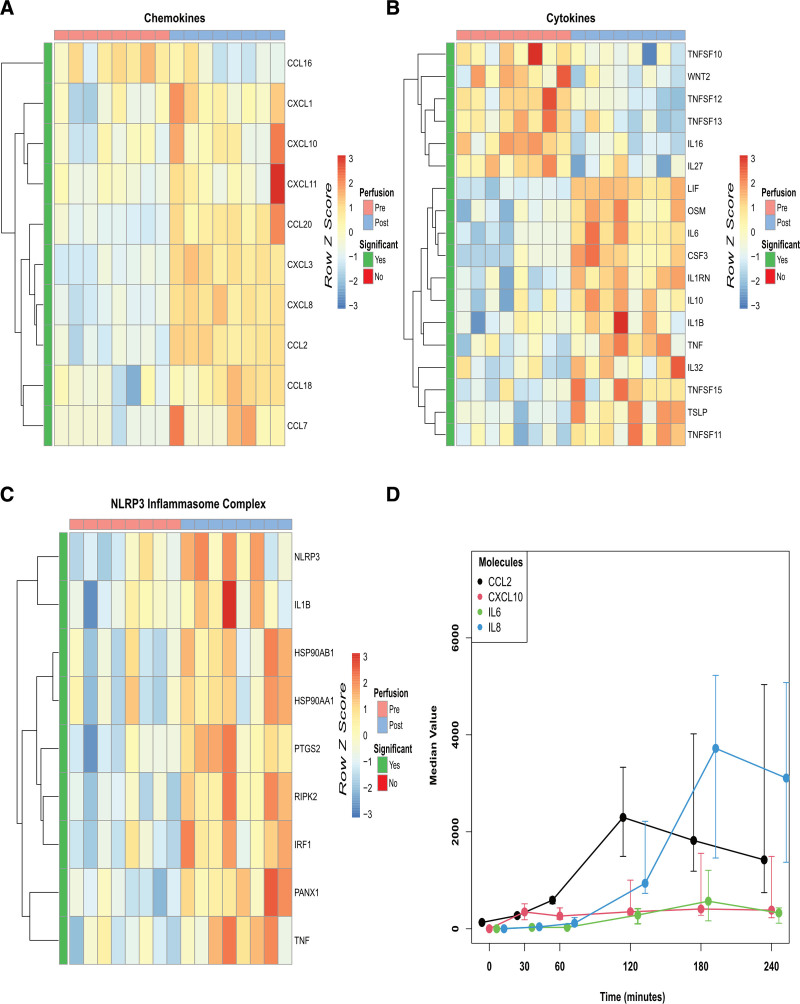
Significant inflammatory changes seen in gene expressions of DCD livers undergoing ESNP. A and B, Heatmap of all human chemokines and cytokines expressed post-ESNP, with genes exhibiting significant differential expression (adjusted *P* < 0.05) highlighted in green and clustered by rows. C, Heatmap of selected, curated inflammasome genes post-ESNP, with genes exhibiting significant differential expression (adjusted *P* < 0.05) highlighted in green and clustered by rows. D, Scatter plot displaying the overall increase in cytokine levels measured in the perfusate up to 240 min of ESNP, with individual cytokines showing varied patterns, including some peaking at 120 min. Error bars represent their respective IQRs. DCD, donation after circulatory death; ESNP, ex situ normothermic perfusion; IQR, interquartile range.

**FIGURE 3.**
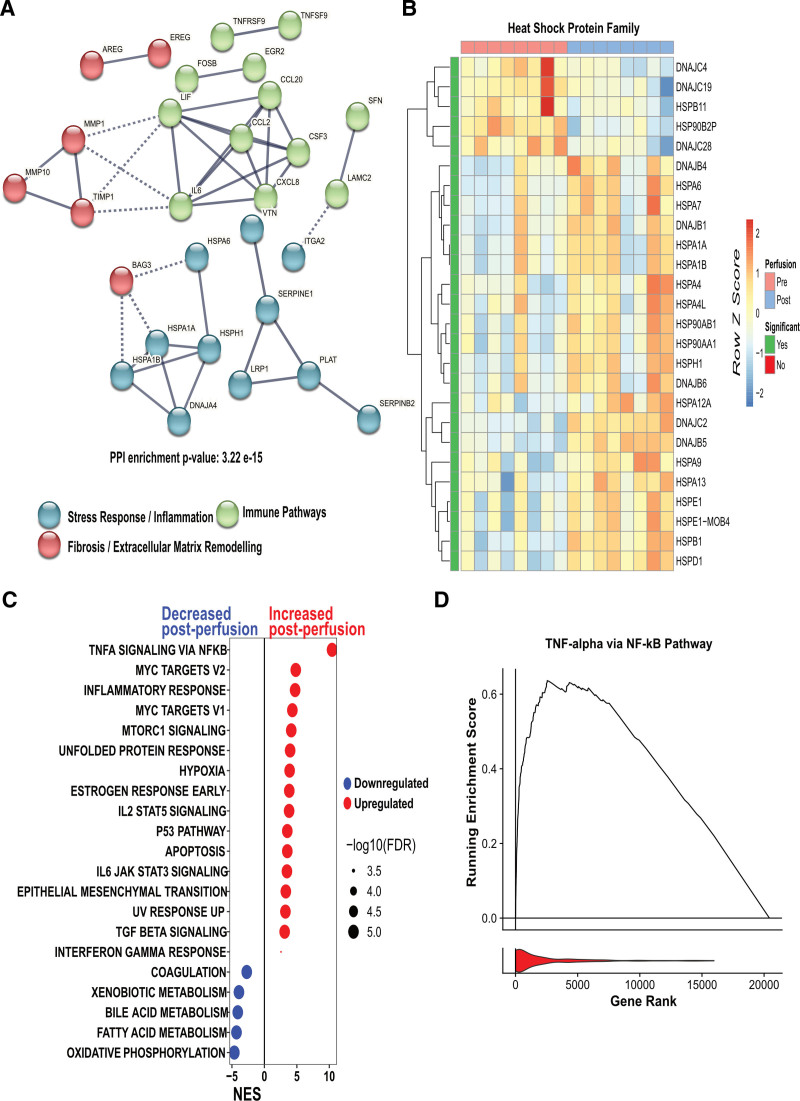
Transcriptomic changes associated with increased inflammatory and fibrotic shifts in DCD livers undergoing ex situ normothermic machine perfusion. A, STRING analysis of the top 100 significantly upregulated genes. B, Heatmap representing the differential expression of all HSPs genes during ESNP. Differentially expressed genes showing significant upregulation (adjusted *P* < 0.05) are highlighted in green and clustered by rows. C, GSEA comparing DCD livers undergoing ESNP against the Hallmark data set. Pathways showing increased activity post-ESNP are highlighted in red (enriched), and those with reduced activity are marked in blue (depleted)—all gene sets exhibit an FDR q-value of <0.05. Pathways are organized along the y-axis, and the NES is displayed on the x-axis. D, RunRESs of the TNF-alpha via NF-κB gene set from the Hallmark data set, comparing pre and postperfusion samples of DCD livers with a leukocyte filter. The increased activity against its gene rank is denoted in red. DCD, donation after circulatory death; ESNP, ex situ normothermic perfusion; FDR, false discovery rate; GSEA, Gene Set Enrichment Analysis; HSP, heat shock protein; NES, normalized enrichment score; RES, running enrichment score.

**FIGURE 4.**
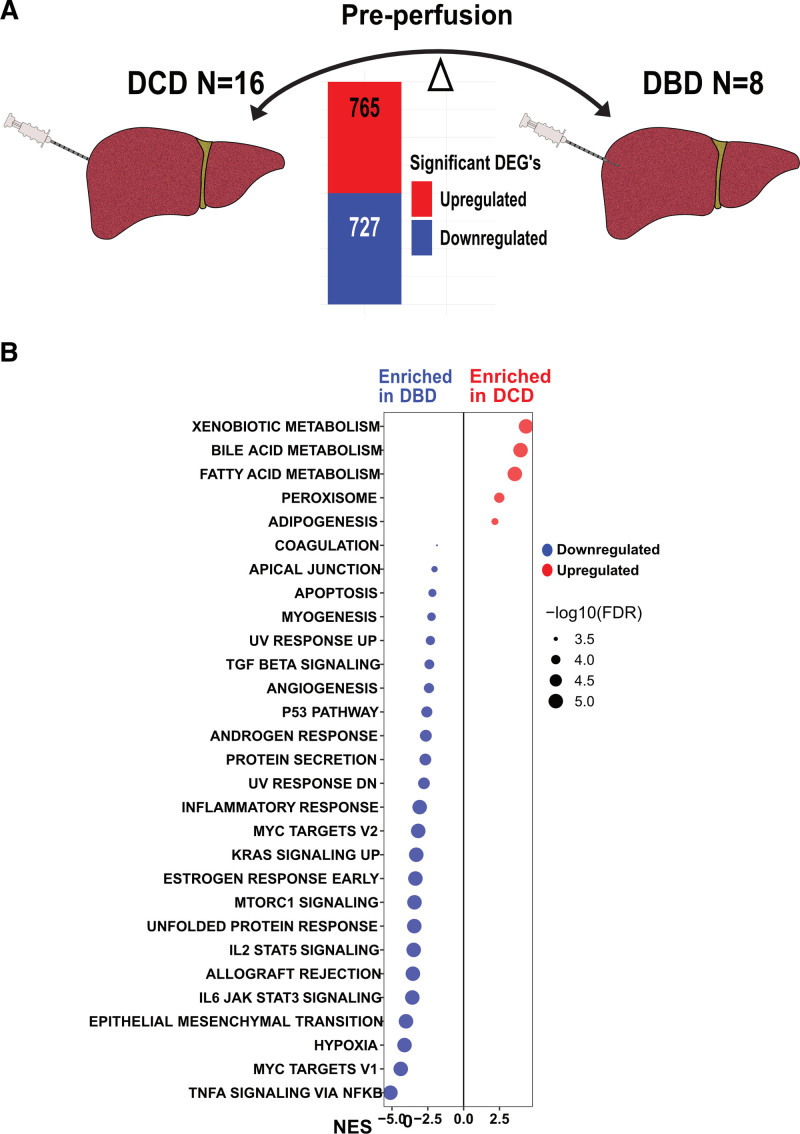
Transcriptional differences observed in DCD and DBD livers before undergoing ESNP. A, Bar graph showing the number of significant DEGs between DCD (n = 16) and DBD (n = 8) livers before ESNP. The red bar represents upregulated genes and the blue bar represents downregulated genes. B, GSEA comparing DCD to DBD livers before undergoing ESNP against the Hallmark data set. Pathways with increased activity in DCD livers are highlighted in red (enriched in DCD), whereas pathways with reduced activity are marked in blue (enriched in DBD). The size of each dot corresponds to the significance of the enrichment, with larger dots representing a lower FDR q-value, all of which are <0.05. Pathways are organized along the y-axis, and the NES is displayed on the x-axis. DBD, donation after brain death; DCD, donation after circulatory death; DEG, differentially expressed gene; ESNP, ex situ normothermic perfusion; FDR, false discovery rate; GSEA, Gene Set Enrichment Analysis; NES, normalized enrichment score.

**FIGURE 5.**
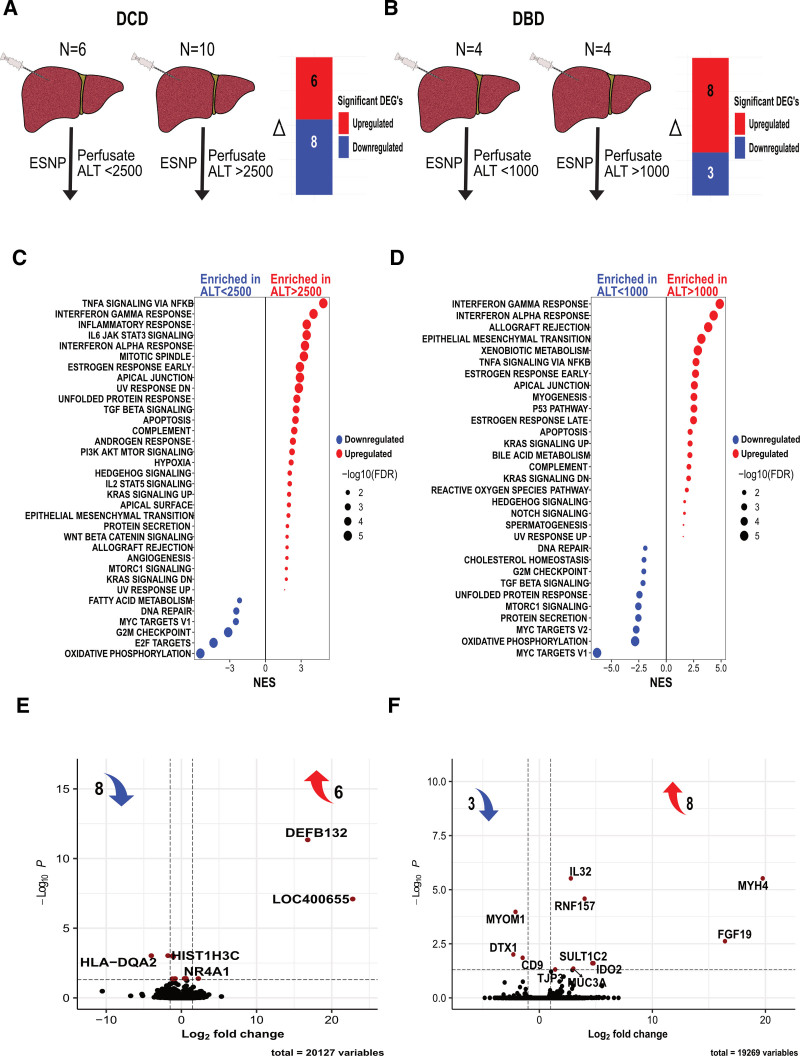
Analysis of transcriptomic profiles and corresponding perfusate ALT levels in DCD and DBD livers during ESNP. A, Bar chart displaying differentially expressed genes comparison of preperfusion samples from DCD livers with a perfusate ALT level >2500 to those with ALT levels <2500. Genes denoted in red indicate upregulation and those in blue represent downregulation. B, Comparison of preperfusion samples from DBD livers with a perfusate ALT level >1000 to those with ALT levels <1000. C and D, GSEA comparing DCD and DBD livers with elevated perfusate ALT levels against the Hallmark data set. Pathways with increased activity seen with increased ALT levels are highlighted in red (enriched in high ALT), and those with reduced activity are marked in blue (enriched in low ALT), with significance defined as an FDR q-value of <0.05. Pathways are organized along the y-axis, and the NES is displayed on the x-axis. E and F, Volcano plots showing changes in gene expression in DCD and DBD livers with elevated perfusate ALT levels, respectively. Genes manifesting significant differential expression (adjusted *P* < 0.05) are marked in red. ALT, alanine transaminase; DBD, donation after brain death; DCD, donation after circulatory death; ESNP, ex situ normothermic perfusion; FDR, false discovery rate; GSEA, Gene Set Enrichment Analysis; NES, normalized enrichment score.

**FIGURE 6.**
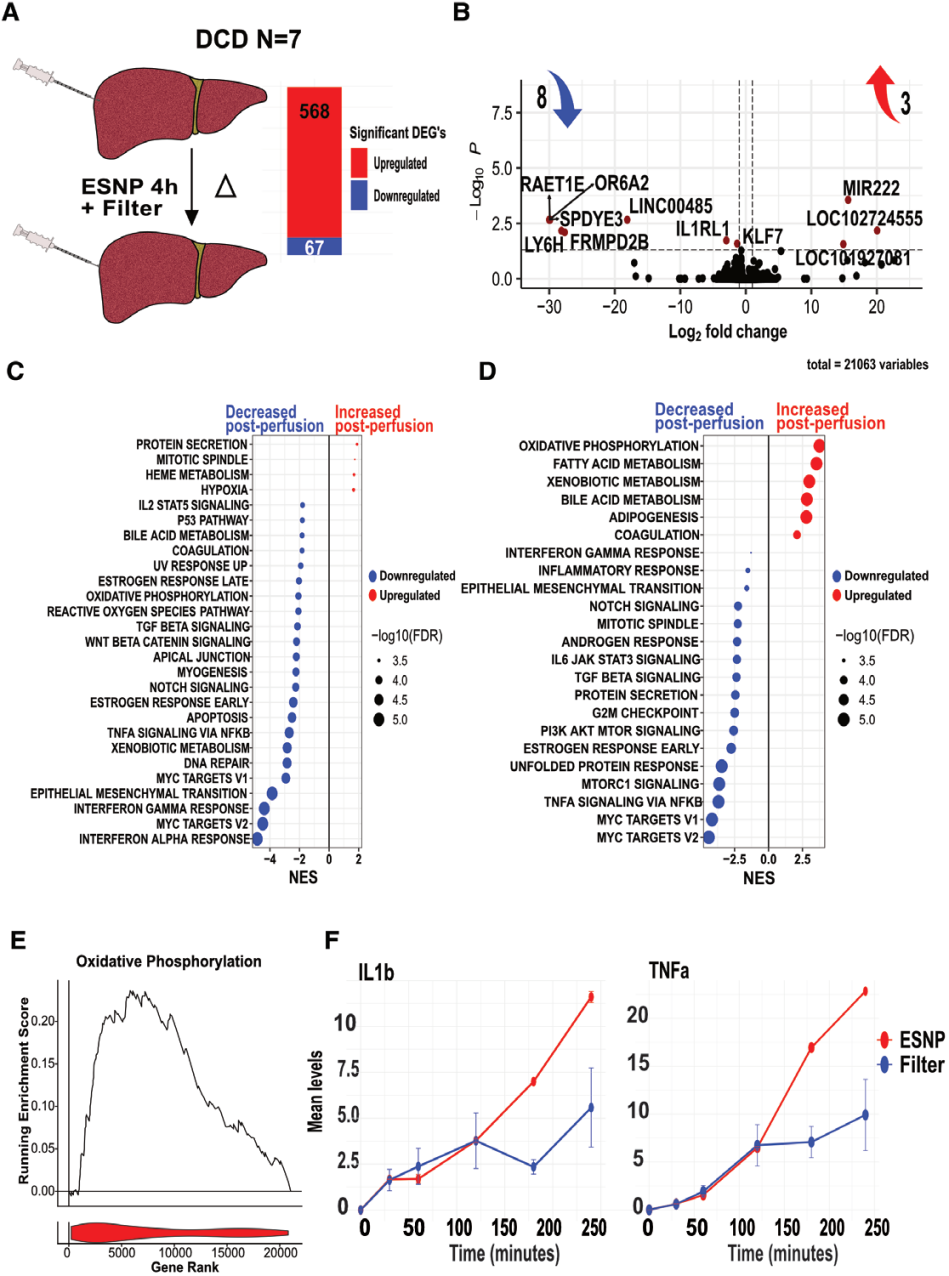
Impact of a leukocyte filter on the transcriptomic profiles during ESNP of DCD livers. A, Bar chart displaying differentially expressed genes in paired pre- and postperfusion samples from 7 DCD livers with the addition of a leukocyte filter. Genes denoted in red indicate upregulation and those in blue represent downregulation. B, Volcano plot showcasing changes in gene expression after 4 h of ESNP with the leukocyte filter compared with ESNP alone as an interaction term (measuring the difference of differences). The most significant (adjusted *P* < 0.05) upregulated and downregulated genes are labeled and marked in red. C, GSEA comparing pre and postperfusion samples from DCD livers during ESNP with a leukocyte filter against the Hallmark data set. Pathways with increased activity post-ESNP are highlighted in red (enriched), and those with reduced activity are indicated in blue (depleted), with significance defined by an FDR q-value <0.05. Pathways are organized along the y-axis, and the NES is displayed on the x-axis. D, GSEA contrasting DCD livers during ESNP with the leukocyte filter as an interaction term against the Hallmark data set. E, RES for the oxidative phosphorylation gene set from the Hallmark data set, comparing pre and postperfusion samples of DCD livers with a leukocyte filter. The increased activity against its gene rank is denoted in red. F, Scatter plot displaying the overall mean increase in cytokine levels of IL-1β and TNF-α measured in the perfusate of livers undergoing ESNP with and without a leukocyte filter >250 min. Both cytokines exhibit a steady increase over time, with a clear reduction observed in samples using a leukocyte filter. Error bars represent the IQR for each time point. DCD, donation after circulatory death; ESNP, ex situ normothermic perfusion; FDR, false discovery rate; GSEA, Gene Set Enrichment Analysis; IL, interleukin; IQR, interquartile range; NES, normalized enrichment score; RES, running enrichment score; TNF-α, tumor necrosis factor alpha.

This study comprised 5 experimental comparisons:

1. Comparative gene expression analysis of paired pre- and post-ESNP DCD liver biopsies. Eight paired samples were available (Figures 1–3; Table 1).
2. Comparative gene expression analysis of pre-ESNP liver biopsies in 16 DCD and DBD liver samples (Figure 4; Tables 1 and 2).
3. Comparative gene expression analysis of preperfusion biopsies in 16 DCD livers, comparing samples with high and low 2-h perfusate alanine transaminase (ALT) levels (Figure 5A, C, E; Table 2).
4. Comparative gene expression analysis of preperfusion biopsies in 8 DBD livers, comparing samples with high and low 2-h perfusate ALT levels (Figure 5B, D, F; Table 2).
5. Comparative gene expression analysis of 7 paired pre- and post-ESNP DCD liver biopsies with the addition of a Pall LeukoGuard LG6 leukocyte filter (Figure 6; Table 3).

**TABLE 1.** Donor liver characteristics livers undergoing ESNP

Donor	Donor age, y	DBD/DCD	Sex (M/F)	Perfusate 2 h ALT	Machine	Lactate concentration (2 h), mmol/L	CIT, h	Transplanted?	Reason not transplanted	Graft survival, d	Patient survival, d	Post-Bx, h
1	35	DCD	M	2648	Liver assist	<0.1	5.4	No	Adverse appearance	NA	NA	4
2	59	DCD	M	4909	Liver assist	0.9	5.1	No	Offered for research only	NA	NA	4
3	76	DCD	M	1573	Liver assist	0.5	10.2	No	Offered for research only	NA	NA	4
4	47	DCD	F	5386	Liver assist	6.8	11.4	No	Offered for research only	NA	NA	4
5	22	DCD	M	4332	Organox Metra	0.8	5.5	Yes	–	1387	1387	11
6	45	DCD	F	3336	Organox Metra	0.6	7.2	No	Bile chemistry	NA	NA	6
7	49	DCD	M	3972	Organox Metra	0.9	6.5	No	Poor function	NA	NA	7
8	55	DCD	M	1361	Organox Metra	0.2	8.9	Yes	–	1057	1057	8

ALT, alanine transaminase; CIT, cold ischemic time; DBD, donation after brain death; DCD, donation after circulatory death; M/F, male/female; NA, not applicable; Post-Bx, timing of biopsy after initiation of perfusion.

**TABLE 2.** Donor liver characteristics (clinical parameters)

Donor	Donor age, y	DBD/DCD	Sex (M/F)	Perfusate 2 h ALT	Machine	Lactate concentration (2 h), mmol/L	CIT, h	Transplanted?	Reason not transplanted	Graft survival, d	Patient survival, d
1	35	DCD	M	2648	Liver assist	<0.1	5.4	No	Adverse appearance	NA	NA
2	47	DCD	F	5386	Liver assist	6.8	11.4	No	Offered for research only	NA	NA
3	59	DCD	M	4909	Liver assist	0.9	5.1	No	Offered for research only	NA	NA
4	68	DCD	M	2658	Organox Metra	0.9	8.5	Yes	NA	360	1804
5	46	DCD	F	4015	Organox Metra	0.4	6.6	Yes	NA	1348	1348
6	22	DCD	M	4332	Organox Metra	0.8	5.6	Yes	NA	1387	1387
7	20	DCD	M	3042	Organox Metra	0.8	3.8	Yes	NA	1575	1575
8	40	DCD	F	3523	Organox Metra	0.9	8.5	Yes	NA	18	1869
9	49	DCD	M	3972	Organox Metra	0.9	6.5	No	Poor function	NA	NA
10	45	DCD	F	3336	Organox Metra	0.6	7.2	No	Bile chemistry	NA	NA
11	76	DCD	M	1573	Liver assist	0.5	8.0	No	Offered for research only	NA	NA
12	66	DCD	M	1799	Liver assist	0.3	7.5	Yes	NA	2511	2511
13	28	DCD	M	2348	Organox Metra	<0.1	6.7	Yes	NA	1324	1324
14	54	DCD	F	1116	Organox Metra	<0.1	6.7	Yes	NA	1801	1801
15	52	DCD	M	1372	Organox Metra	0.2	5.8	Yes	NA	1340	1340
16	55	DCD	M	1361	Organox Metra	0.2	8.9	Yes	NA	1057	1057
17	53	DBD	F	1603	Organox Metra	<0.1	7.0	Yes	NA	1723	1723
18	61	DBD	M	1840	Organox Metra	<0.1	8.2	Yes	NA	57	57^*a*^
19	23	DBD	M	2212	Organox Metra	0.7	9.6	Yes	NA	2	2^*a*^
20	63	DBD	M	1906	Liver assist	0.8	5.9	Yes	NA	2234	2234
21	81	DBD	M	346	Liver assist	<0.1	7.8	No	Offered for research only	NA	NA
22	27	DBD	M	199	Organox Metra	<0.1	7.7	Yes	NA	1891	1891
23	37	DBD	F	639	Organox Metra	<0.1	5.1	Yes	NA	1782	1782
24	28	DBD	F	528	Organox Metra	<0.1	12.1	Yes	NA	1485	1485

a Patient died.

ALT, alanine transaminase; CIT, cold ischemic time; DBD, donation after brain death; DCD, donation after circulatory death; M/F, male/female; NA, not applicable.

**TABLE 3.** Donor liver characteristics (addition of leukocyte filter)

Donor	Donor age, y	DBD/DCD	Sex (M/F)	Perfusate 2 h ALT	Machine	Lactate concentration (2 h), mmol/L	CIT, h	Transplanted	Reason not transplanted	Graft survival, d	Patient survival, d	Post–Bx, h
1	66	DCD	F	2439	Liver assist	10.1	8.7	No	Offered for research only	NA	NA	4
2	68	DCD	M	3623	Liver assist	0.7	12.8	No	Offered for research only	NA	NA	4
3	69	DCD	M	4088	Liver assist	3.6	11.2	No	Offered for research only	NA	NA	4
4	48	DCD	F	694	Liver assist	<0.1	8.0	No	Offered for research only	NA	NA	4
5	28	DCD	F	216	Liver assist	<0.1	4.9	Yes	NA	2487	2487	4
6	54	DCD	M	262	Liver assist	<0.1	7.3	Yes	NA	2478	2478	4
7	69	DCD	F	1631	Liver assist	0.9	5.5	Yes	NA	2367	2367	5

ALT, alanine transaminase; CIT, cold ischemic time; DBD, donation after brain death; DCD, donation after circulatory death; M/F, male/female; NA, not applicable; Post-Bx, timing of biopsy after initiation of perfusion.

### Perfusion Analysis Based on ALT Levels

Perfusate ALT levels were measured 2 h after the start of perfusion for 16 DCD and 8 DBD livers (Table 2). The thresholds that were used to assign livers into “high” and “low” ALT groups were established on the basis of the level at which increased inflammatory and fibrotic gene expression pathways were observed. In DBD livers, this occurred at an ALT level of 1000 IU/L. For DCD livers, this was evident at an ALT level of 2500 IU/L (see **Figure S1B, SDC,** http://links.lww.com/TP/D167). Because of the difference in donor type and exposure to warm ischemia, we opted to have separate thresholds for DCD and DBD rather than using a universal cutoff.

### RNA Extraction

RNA was extracted from biopsies stored in RNALater (Ambion) at –80 °C. Biopsies were removed from their storage solution and placed with 1 mL Lysis Buffer (Ambion) in an MK28-R grinder tube (Bertin Instruments) and lysed using a Precellys 24 homogenizer (Bertin Instruments). Tubes were centrifuged at 1500*g* for 4 min, the supernatant removed, and the RNA extraction performed using a pure link RNA mini kit (Ambion) as per manufacturer’s instructions. Contaminating DNA was removed using TURBO DNase (Ambion) as per manufacturer’s instructions. The concentration of RNA was assessed using a Nanodrop Spectrophotometer (Thermo Scientific). The quality of RNA was assessed using an RNA nano Bioanalyzer kit (Agilent) using a Bioanalyzer 2100 (Agilent).

### RNA Sequencing

An RNA concentration of 0.5 µg was used to produce sequencing libraries using TruSeq Stranded Total RNA Library preparation kit (Illumina) as per manufacturer’s instructions with final polymerase chain reaction amplification of 14 cycles. Libraries were then sequenced on a Hiseq 4000 sequencer (Illumina) by Genewiz.

### RNA Sequencing Analysis

After sequencing, data were demultiplexed to give individual Fastq files using Casava (Illumina). Fastq files were assessed for quality control purposes using FASTQC. The Fastq files were aligned to the human genome (Hg38) using Hisat216. All further analyses were carried out using the R statistical environment. A table of gene counts was produced using the featureCounts function within Rsubread and normalization, and differential gene expression analysis was carried out using DESeq2. For Gene Set Enrichment Analysis (GSEA), genes were ranked by the inverse of the *P* value with the sign of the log fold change and then run against the Hallmark database within Molecular Signatures Database using the GSEA program from the Broad Institute with the preranked option. All batch information, Fastq files, count tables, and differential expression results have been submitted to the Gene Expression Omnibus under accession number GSE245976.

### Data Visualization

The ggplot2 package in R was used for data visualization. This enabled the creation of various plots and heatmaps using pheatmap to analyze the distribution of normalized gene expression values across samples or conditions and assess variance within our data set. Differential gene expression results were visualized through Volcano plots using the EnhancedVolcano package. Custom modifications were applied to highlight and annotate genes of significance based on fold change and *P* value thresholds. Further refinements in visualization aesthetics were performed using ggplot2.

Complete code and further details for all visualizations are available upon request.

### String Analysis

The top 100 significant genes, ranked by log fold change for the effect of perfusion, were used to run a STRING analysis of functional associations between proteins. Gene names were converted into protein names and 2 proteins were considered connected if they had a mean interaction score of >0.9 (highest confidence). Unconnected proteins were removed from the network, and a force-directed graph was plotted. The network was subsequently clustered using k-means clustering and annotated using an interpretation of gene ontology terms associated with each cluster.

## RESULTS

### ESNP Associated With Increased Expression of Clotting Factor and Regulator Genes

Eight DCD livers (see Table 1 for donor characteristics) underwent ESNP as described previously.^6^ When comparing the 2 time points, we found a substantial change in the transcriptome of DCD livers during perfusion, with >3600 differentially expressed genes (DEGs), including ~2100 that were significantly upregulated and ~1550 significantly downregulated (Figure 1A).

The 2 most significantly upregulated genes during ESNP were *SERPINE1* and *THBS1* (Figure 1B). *THBS1* encodes Thrombospondin 1, a protein that promotes thrombin-induced platelet aggregation.^15^ *SERPINE1* encodes plasminogen activator inhibitor-1 (PAI-1), a member of the serine proteinase inhibitor superfamily and the principal inhibitor of tissue plasminogen activator (tPA) and urokinase, both mediators of fibrinolysis through plasmin (Figure 1C). To probe this further, we assessed the expression of genes encoding clotting factors and regulators of thrombosis and fibrinolysis. Comparing post- with preperfusion samples, several pro- and antithrombotic/fibrinolytic proteins were significantly differentially expressed (Figure 1C and D), including increased expression of *SERPINB2*, encoding PAI-2, which also acts as an inhibitor of tPA and urokinase. However, *PLAU* and *PLAT* (encoding urokinase and tPA, respectively) expression also increased during ESNP (Figure 1C and D), although to a lesser magnitude than *SERPINE1*. Interestingly, despite the observed alterations in the expression of several thrombotic and fibrinolytic genes postperfusion, the levels of plasminogen (*PLG*) and the fibrinogen chain genes (*FGA, FGB, and FGG*) were downregulated, albeit not to a statistically significant level.

### Increased Expression of Neutrophil and Monocyte-recruiting Chemokines and Prorepair Transcripts

A number of immunologically important transcripts were also among the most upregulated genes in DCD livers during ESNP, including the neutrophil recruiting chemokine *CXCL8*, the monocyte-recruiting chemokine *CCL2*, and the proinflammatory cytokine interleukin *(IL)6* (Figure 1B). Several other chemokine (Figure 2A) and cytokine (Figure 2B) transcripts were also significantly upregulated during ESNP, including *CXCL10*, *TNF*, *IL1B*, and *OSM*, the latter encoding oncostatin-M, a cytokine previously shown to drive liver fibrosis by activating macrophages to secrete transforming growth factor-β in murine models.^16^

There was also an increase in key inflammasome genes (*NLRP3* and *P2RX7*) in DCD livers after perfusion (Figure 2C). Sequential assessment of perfusate samples during ESNP confirmed an increase in CXCL8 (interleukin [IL]8), CXCL10, CCL2, IL6, and IL-1β proteins (Figure 2D).

We next used STRING analysis to interrogate known and predicted interacting proteins among upregulated transcripts. This identified several interaction nodes, including an immune node, with *IL6*, *CXCL8*, *CCL20*, and *CSF3* (encoding GCSF, a cytokine that promotes neutrophil production and maturation). Other immune nodes included *TNFRSF9-TNFSF9* (encoding 4-1BB and 4-1BB-L, a costimulatory axis driving T-cell proliferation, IL-2 secretion, and survival and cytolytic activity; Figure 3A). There were also nodes comprising genes associated with tissue remodeling (*MMP1/10, TIMP1*) and repair (*AREG/EREG*; Figure 3A).

There was an increase in several additional potential prorepair or protective transcripts, including *TSLP*, encoding thymic stromal lymphopoietin (TSLP), an epithelial-derived cytokine (Figure 2B). Administration of exogenous recombinant TSLP significantly reduced liver damage in a mouse model of IRI, whereas deletion of TSLP receptor or neutralization of TSLP with anti-TSLP antibody exacerbated liver injury and necrosis.^17^ In addition, several heat shock proteins (HSPs) also significantly increased during ESNP (Figure 3B), including *HSPB1* (*HSP27*), the overexpression of which has been shown to protect the murine kidney, heart, and liver cells from apoptosis in the context of IRI.^18,19^

GSEA also indicated increases in both immune/inflammatory and repair/proliferation pathways during ESNP. Enriched immune pathways included “tumor necrosis factor alpha (TNF-α) signaling via nuclear factor kappa B (NF-κB),” “inflammatory response,” “IL2/signal transducer and activator of transcription 5 signaling,” “IL6/JAK/signal transducer and activator of transcription 3 signaling” gene sets, together with those indicative of cell proliferation, (Myc genes) and “epithelial-mesenchymal transition” (Figure 3C). Decreased enrichment of “oxidative phosphorylation” (OXPHOS) pathway genes, which play a crucial role in cellular ATP generation,^20^ was observed in DCD livers during ESNP, as well as “bile acid metabolism” gene sets (Figure 3C).

### DBD and DCD Livers Show Transcriptional Differences

ESNP has principally been used in DCD livers, but we also applied perfusion to 8 DBD livers based on suboptimal clinical profiles (see Table 2). Preperfusion biopsies were available on all 8 DBD livers, although no postperfusion samples. We, therefore, compared the baseline gene expression in these DBD livers with pre-perfusion biopsies in 16 DCD livers (Figure 4A). GSEA showed enrichment of several metabolic gene pathways in DCD livers, including “fatty acid metabolism,” whereas several immune-related pathways were enriched in DBD livers, as well as “epithelial-mesenchymal transition” pathway genes (Figure 4B). These data show that DBD and DCD livers are transcriptionally distinct postretrieval and cold storage, likely reflecting the effects of increased warm ischemia in DCD donors.

### Molecular Correlates of Perfusion and Clinical Parameters

To investigate transcriptional features that might be associated with unfavorable outcomes, we assessed the gene expression profile present in preperfusion biopsies taken from livers that subsequently had an elevated perfusate ALT concentration, a biomarker associated with hepatocyte injury,^21^ and previously identified by us as a key marker of “viability.”^22^

Perfusate ALT levels measured 2 h postperfusion were available for 16 DCD and 8 DBD livers that had undergone ESNP (Table 2; Figure 5A and B). In DCD livers, GSEA comparing 10 livers with ALT levels >2500 IU/L with 6 livers with ALT levels <2500 IU/L revealed significant enrichment of immune activation gene sets, including “TNF-α signaling via NF-κB,” “interferon-gamma response,” and “inflammatory response” (Figure 5C). For DBD livers, where perfusate ALT levels were generally lower, the gene sets “interferon-gamma response,” “interferon-alpha response,” and “allograft rejection” were significantly enriched in livers with ALT levels exceeding 1000 IU/L (Figure 5D). Additionally, both DCD and DBD livers with higher ALT levels showed a marked decrease in “oxidative phosphorylation” genes (Figure 5C and D).

Only 16 genes were significantly differentially expressed in DCD livers with a higher perfusate ALT level (>2500) compared with those with a lower ALT level (<2500; Figure 5E). Upregulated genes included *DEFB132* (encoding the antimicrobial peptide defensin beta 132), the uncharacterized gene *LOC400655*, and *NR4A1* encoding nuclear receptor subfamily 4A1, a transcription factor expressed in Kupfer cells and implicated in a caspase6/SOX9 signaling axis associated with hepatic inflammation and pyroptosis in ischemic fatty livers.^23^ Eleven genes were significantly differentially expressed in DBD livers with a high perfusate ALT (Figure 5F). Among those, *IL32* and *FGF19* were significantly upregulated. IL32 is of particular interest, given its recent description as the most upregulated transcript in severe metabolic dysfunction-associated steatotic liver disease livers compared with controls, with circulating levels of IL32 associated with both the presence and severity of metabolic dysfunction-associated steatotic liver disease independently of ALT.^24^ Similarly, fibroblast growth factor 19 (FGF19) mRNA expression was 7-fold greater in cirrhotic livers compared with noncirrhotic livers, with increased liver and serum levels of FGF19 also noted in these patients with primary biliary cholangitis. FGF19 acts to inhibit the synthesis of potentially toxic bile acids.^25,26^ Taken with the literature on the roles of these molecules outside of the transplant context, our results highlight the potential for IL32 and FGF19 as biomarkers of suboptimal DBD livers.

### Leucocyte Removal Attenuates Perfusion-associated Transcriptional Changes

Several factors may contribute to the increase in inflammation and immune-related gene sets we observed during ESNP, including the release of proinflammatory cell damage-associated molecules that can further exacerbate the induction of inflammatory genes (as we have demonstrated in the kidney^9^). In addition, leucocytes trapped within the graft microcirculation, as well as tissue-resident immune cells, may mobilize into the perfusion circuit.^27^ These include neutrophils and monocytes, with the capacity to propagate inflammation and cause collateral damage.^13^ We therefore hypothesized that removal of leucocytes from the perfusion circuit may attenuate the transcriptional changes occurring during perfusion. To test this, we added a leucocyte filter to the arterial limb of the perfusion circuit in 7 DCD livers, sampling pre- and post-ESNP (Figure 6A; Table 3). The reduction in circulating leukocytes reduced perfusion-associated transcriptional changes, with a 6-fold decrease in the number of DEG compared with DCD livers undergoing ESNP without a leucocyte filter (~600 DEG compared with ~3600; Figure 6A). Furthermore, the addition of a leucocyte filter changed the nature of the gene set enrichment occurring during perfusion compared with ESNP alone, leading to a decrease in “TNF-α signaling via NF-κB,” “epithelial-mesenchymal transition,” and “interferon-gamma and alpha response,” pathway genes (Figure 6C). When comparing the DEG between ESNP with and without a leucocyte filter, only 11 genes were significantly differentially expressed, including downregulation of *RAET1E* (encoding an NKG2D ligand that can trigger natural killer cell–mediated cytotoxicity)^28^ and *IL1RL*, a component of the receptor for IL33, a proven driver of liver fibrosis in mouse models (Figure 6B).^29^ There was a significant increase in OXPHOS and several metabolism-associated gene sets, and a decrease in “TNF-α signaling via NF-κB,” “transforming growth factor beta signaling,” and “epithelial-mesenchymal transition” gene sets in livers undergoing ESNP with a leukocyte filter compared with those without (Figure 6D and E). Consistent with an anti-inflammatory effect of leucocyte removal, the level of TNF and IL1B was lower in perfusate samples in the presence of a cell filter (Figure 6F; **Figure S2, SDC,** http://links.lww.com/TP/D167).

## DISCUSSION

The common reasons that livers are declined for transplantation are the unpredictability of function and high incidence of cholangiopathy of DCD donor livers, concern over steatotic livers with their severe reperfusion injury, and higher incidence of primary nonfunction. Thus, to increase the utilization of donor livers, there is a strong imperative to understand the molecular events occurring during liver reperfusion in vivo, for which reperfusion on ESNP is a model, with a view to developing treatments that can ameliorate or prevent reperfusion injury, improve early allograft function, and prevent cholangiopathy.

One of the most upregulated genes during ESNP was *SERPINE1*, with *SERPINB2* also showing a significant increase, which encodes PAI-1 *and* PAI-2, respectively. These proteins act as to inhibit tPA and urokinase, the 2 serine proteases required for the conversion of plasminogen to plasmin, a key fibrinolytic. Increased expression of PAI1 has been associated with fibrin accumulation in hypoxic lungs in mice^30^ and may mechanistically underpin the peribiliary vascular fibrin microthrombi described in livers undergoing ESNP.^31,32^ We also observed an increase in profibrinolytic factor expression, specifically *PLAU* and *PLAT* encoding urokinase and tPA, respectively, although to a lesser magnitude and significance than that observed for *SERPINE1*. Whether such differences in the magnitude of transcriptional changes of these profibrinolytics and antifibrinolytics are mirrored at the protein level is an outstanding question that needs to be addressed to assess their functional relevance, and whether the net effect of the changes occurring during ESNP promotes the persistence of fibrin microthrombi. We did not have access to preretrieval biopsies and were therefore unable to assess whether the levels of these genes were upregulated after warm ischemia or during cold storage.

We have identified the upregulation of several immune genes that may contribute to the inflammatory response that characterizes reperfusion injury during ESNP. Predominant among these were *CXCL8* and *CCL2*, recruiting neutrophils and monocytes, respectively, and *IL6*. The latter observation is of particular interest given a recent study suggesting that the transcriptional profile of neutrophils changes during liver perfusion toward an activated or exhausted state.^13^ The increase in these transcripts was also reflected at a protein level when measured in the liver perfusate. Altogether, these findings are consistent with observations by Hautz et al,^13^ who reported neutrophil and monocyte mobilization into the perfusate during normothermic machine perfusion, as well as high *IL6* levels, particularly in less-than-ideal grafts, such as those from DCD donors. Our analysis also identified a significant upregulation of GCSF, the cytokine known to drive neutrophil maturation and mobilization from the bone marrow. This local production of GCSF within the liver may contribute to the change in neutrophil maturation/activation state occurring during liver perfusion, described by Hautz and colleagues.^13^ Additionally, the *NLRP3* inflammasome plays a critical role in the inflammatory response during ESNP by acting as a major hub to generate IL-1β in response to damage-associated molecular patterns.^33^ Indeed, perfusate IL-1β has been proposed as a potential prognostic biomarker in lungs undergoing ESNP.^34^ Notably, we found that the addition of a leucocyte filter to the perfusion circuit reduced perfusate IL-1β and TNF. TNF has been shown to inhibit oxidative phosphorylation via effects on cytochrome c oxidase^35^ and IL1R signaling reprogrammes metabolic activity in some cell types, suppressing oxidative phosphorylation and fatty acid oxidation.^36^ Therefore, the reduction of these cytokines in perfusate in livers perfused with a cell filter may be of direct mechanistic relevance to the observed increase in OXPHOS and fatty acid oxidation genes observed in these livers.

In the DCD livers perfused without a cell filter, OXPHOS pathway genes decreased during perfusion despite the provision of oxygen in the perfusate. One explanation may be that during cold ischemia, purine nucleotides are degraded such that there is no longer substrate from which to generate ATP.^37^ Ischemia also causes the accumulation of succinate at complex 1 of the respiratory chain of mitochondria and on reperfusion this promotes the production of reactive oxygen species, which destroy the mitochondria, reducing the ability for oxidative phosphorylation.^38^ Another possibility relates to an observation that we reported previously and have encountered several times since, where the perfusate at the start of ESNP has a glucose concentration <10 mmol/L, in contrast to most livers where the glucose is high and rises further during the first 1 to 2 h before falling.^39^ A possible explanation for this phenomenon is that patients in Neurosurgical Intensive Care Units who do not tolerate enteral feeding do not receive supplementary parenteral nutrition for the first few days of their admission because this is deleterious to neurological recovery. As a consequence, hepatic glycogen stores are depleted, such that during cold storage, there is little or no available glucose with which to generate ATP to maintain cell viability.^37^

One of the challenges in assessing livers undergoing ESNP is to identify those livers at risk of primary nonfunction and early graft failure. In our previous analysis of parameters during liver ESNP, we identified transaminase release and lactate metabolism as 2 key factors.^22^ Transcriptomic analysis revealed that livers sustaining more hepatocellular damage during cold storage and reperfusion, as demonstrated by the more elevated ALT levels at 2 h, showed enrichment of interferon-gamma response pathway genes, but a decrease in expression of oxidative phosphorylation genes, the latter possibly secondary to mitochondrial dysfunction/destruction and associated cytolysis, as indicated by the transaminase release. Among individual genes that were significantly differentially expressed in high ALT livers, we identified a number of putative mechanistic biomarkers that will require further study, including *IL32* and *FGF19* in DBD livers and *NR4A1* in DCD livers, supported by orthogonal literature on their role in liver IRI or fibrosis.^24–26^

Our study has a number of limitations. First, the number of livers and samples analyzed in this study, while providing valuable initial insights, is relatively small. Second, the timing of sampling the liver and perfusate was variable, which may have caused some variation in quantitative changes in gene expression, although it is likely that there is no qualitative difference. Third, we do not have tissue protein analysis to support transcriptomic observations. Finally, as ESNP became the standard of care for marginal livers in our practice and for donor and logistic indications, we changed from Liver Assist to the Organox Metra ESNP device. The main differences in liver perfusion are that the perfusate volume was 500 mL greater in the Liver Assist, and an infusion of taurocholic acid bile salts was given on the Organox Metra. However, we did not compare the differential effects of these 2 devices on the transcriptional changes occurring during ESNP because of the substantial confounding introduced by the change in selection practices for ESNP livers over time.

In summary, our work defines the molecular changes occurring during ESNP. It demonstrates a substantial shift in the transcriptome of DCD livers during reperfusion after cold storage, with the induction of several potentially proinflammatory genes, chemokines, and tissue remodeling/repair genes, as well as the generation of a potentially antifibrinolytic state. It also highlights the potential therapeutic benefits of including a leukocyte filter within the perfusion circuit. The use of such filters could mitigate some of the adverse inflammatory responses observed, offering a more stable environment that promotes repair and regeneration, ultimately enhancing the viability and function of the graft.

## Supplementary Material


