# Supplementary material for: Inflammatory Gene Expression in Livers Undergoing Ex Situ Normothermic Perfusion Is Attenuated by Leukocyte Removal From the Perfusate
Source: Transplantation. 2025 Jan 20;109(2):332–45. doi: 10.1097/TP.0000000000005214 (PMC11745667; doi:10.1097/TP.0000000000005214)
Supplement: Supplementary file 1 [file tpa-109-0332-s001.pdf]

Figure S1

A

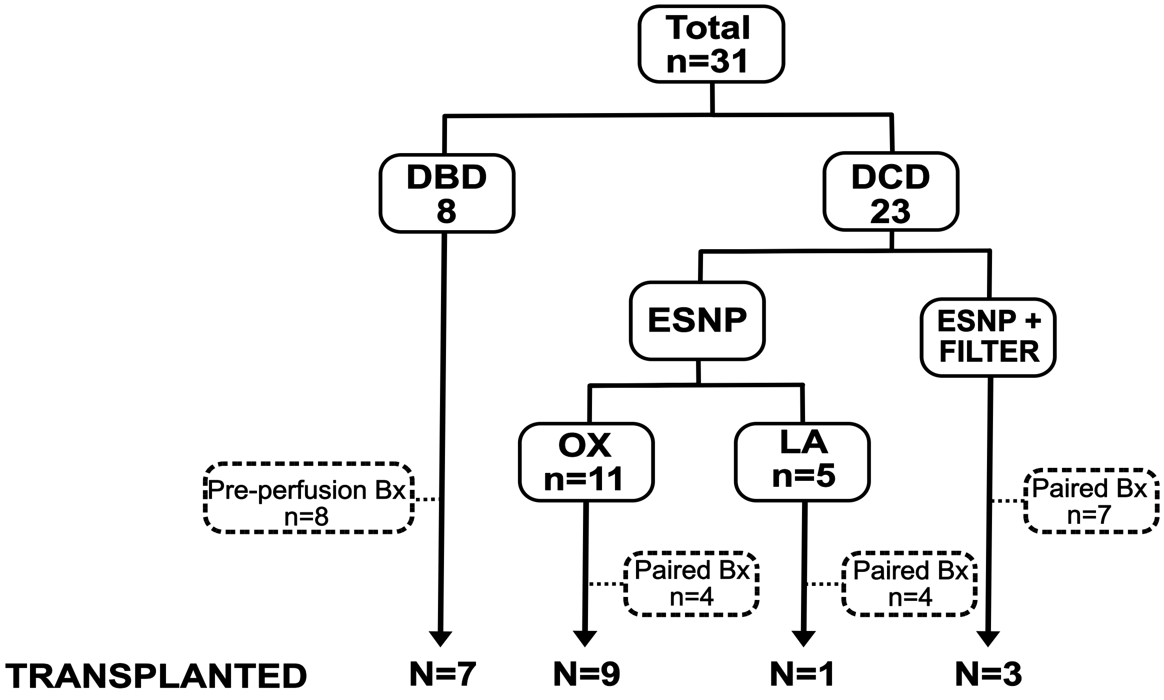

Key: DBD: Donation after brain death; DCD: Donation after circulatory death; ALT: Alanine transaminase; OX: Organox Metra; LA: Liver Assist; ESNP: Ex-situ normothermic perfusion; FILTER: leukocyte filter

B

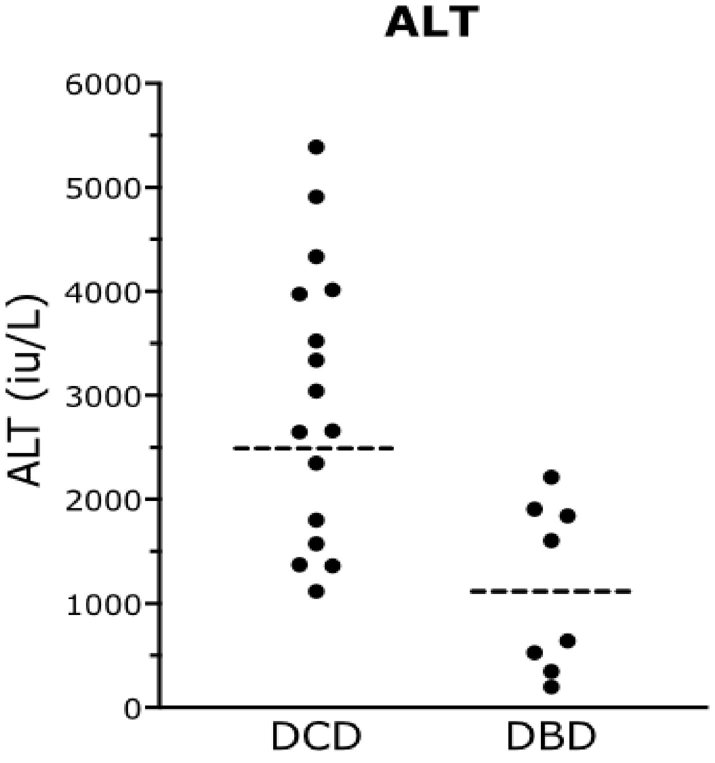

Figure S2

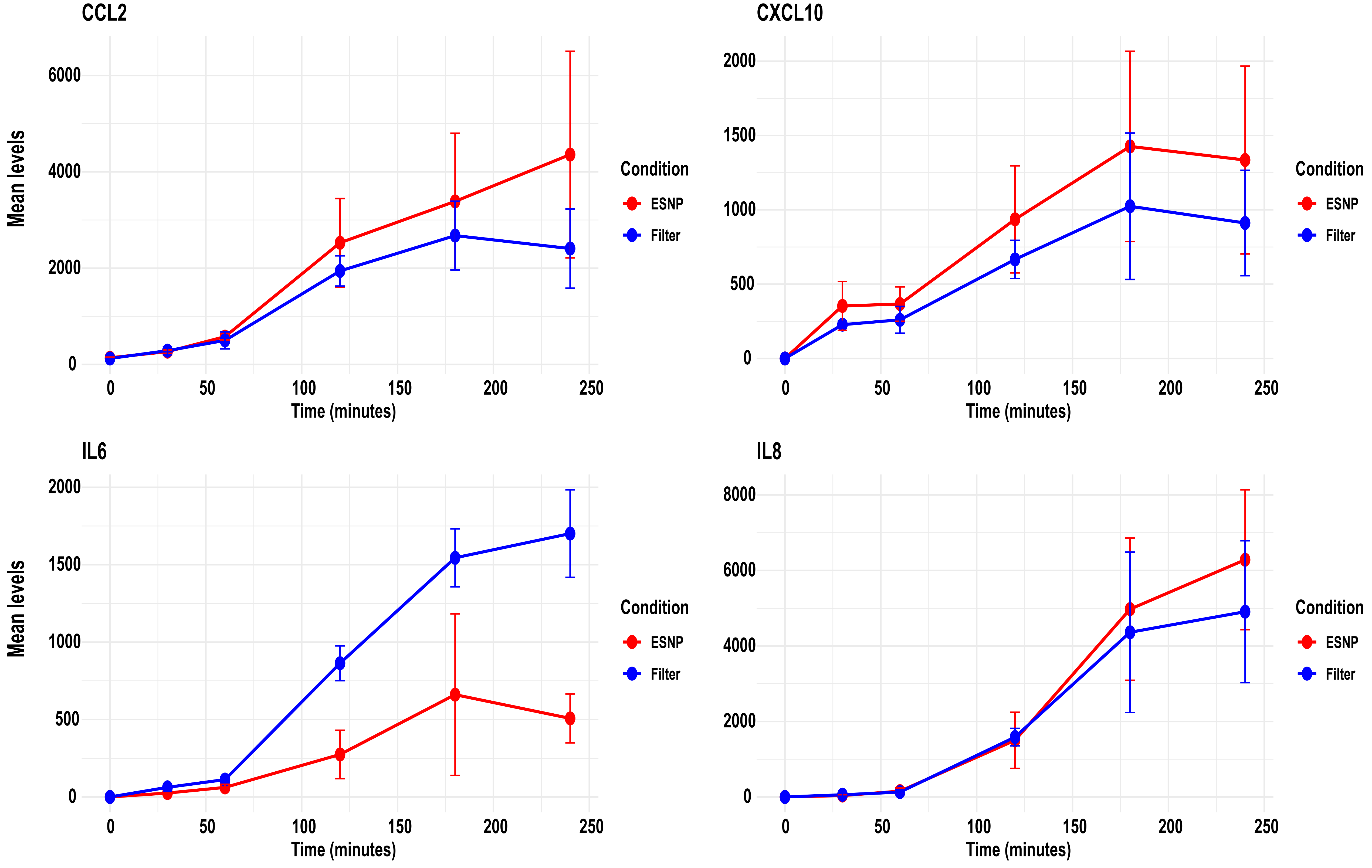
CCL2
Mean levels
6000
4000
2000
0
0
50
100
150
200
250
Time (minutes)
Condition
ESNP
Filter
CXCL10
2000
1500
1000
500
0
IL6
2000
1500
1000
500
0
IL8
8000
6000
4000
2000
0
